# ChIP-BIT2: a software tool to detect weak binding events using a Bayesian integration approach

**DOI:** 10.1186/s12859-021-04108-5

**Published:** 2021-04-15

**Authors:** Xi Chen, Xu Shi, Andrew F. Neuwald, Leena Hilakivi-Clarke, Robert Clarke, Jianhua Xuan

**Affiliations:** 1 Bradley Department of Electrical and Computer Engineering, Virginia Polytechnic Institute and State University, 900 North Glebe Road, Arlington, VA 22203 USA; 2 Center for Computational Biology, Flatiron Institute, Simons Foundation, 162 Fifth Avenue, New York, NY 10010 USA; 3 Institute for Genome Sciences and Department Biochemistry and Molecular Biology, University of Maryland School of Medicine, Baltimore, MD 21201 USA; 4 Hormel Institute, University of Minnesota, 801 16th Ave NE, Austin, MN 55912 USA

## Abstract

**Background:**

ChIP-seq combines chromatin immunoprecipitation assays with sequencing and identifies genome-wide binding sites for DNA binding proteins. While many binding sites have strong ChIP-seq ‘peak’ observations and are well captured, there are still regions bound by proteins weakly, with a relatively low ChIP-seq signal enrichment. These weak binding sites, especially those at promoters and enhancers, are functionally important because they also regulate nearby gene expression. Yet, it remains a challenge to accurately identify weak binding sites in ChIP-seq data due to the ambiguity in differentiating these weak binding sites from the amplified background DNAs.

**Results:**

ChIP-BIT2 (http://sourceforge.net/projects/chipbitc/) is a software package for ChIP-seq peak detection. ChIP-BIT2 employs a mixture model integrating protein and control ChIP-seq data and predicts strong or weak protein binding sites at promoters, enhancers, or other genomic locations. For binding sites at gene promoters, ChIP-BIT2 simultaneously predicts their target genes. ChIP-BIT2 has been validated on benchmark regions and tested using large-scale ENCODE ChIP-seq data, demonstrating its high accuracy and wide applicability.

**Conclusion:**

ChIP-BIT2 is an efficient ChIP-seq peak caller. It provides a better lens to examine weak binding sites and can refine or extend the existing binding site collection, providing additional regulatory regions for decoding the mechanism of gene expression regulation.

**Supplementary Information:**

The online version contains supplementary material available at 10.1186/s12859-021-04108-5.

## Introduction

ChIP-seq technique combines chromatin immunoprecipitation (ChIP) assays with massively parallel sequencing (Seq) and delivers genome-wide profiling of DNA sites bound by a specific protein [1, 2]. DNA-associated proteins mainly include transcription factors (TFs) and histone modification proteins (HMs) and they have diverse functional roles in the epigenome. Master TFs [3] bind at specific DNA locations and most have strong ChIP-seq signal enrichment [4]. Partner TFs and most HMs bind at more diverse loci and some of them have weak ChIP-seq signal enrichment at long DNA segments [5–7]. All generate mechanistically important regulatory actions on nearby gene transcription. Yet, accurately identifying the weak binding sites is challenging because their relatively low signals in the ChIP-seq experiment can be easily obscured by the noise signals produced by the amplified background DNAs.

The ChIP-BIT algorithm (Bayesian inference of target genes using ChIP-seq data) was developed by Chen et al. and was originally applied to detecting the narrow TF binding sites (TFBSs) near to the gene transcription starting sites (TSSs) and predicting target genes [8]. Using a multi-component mixture distribution to jointly model ChIP-seq read intensities in the sample (protein) and the input ChIP-seq experiments, ChIP-BIT can better capture weak peaks and predict their target genes. Recent research on distal regulatory regions like enhancers has demonstrated the functional importance of protein binding sites at these regions on distal gene regulation [9]. Proteins like EP300, H3K27ac, and H3K4me1 bind to enhancers more frequently than at promoters [10–12]. For ChIP-seq data of such proteins, the peak detection capability of ChIP-BIT is very limited. Moreover, many HMs are having very wide peaks crossing thousands of base pairs [13]. Compared to the narrow and sharp ChIP-seq peaks, ChIP-seq signals of wide peaks are not central to the peak summits but spreading along wide genome segments. These wide peaks are also out of the peak width scope that ChIP-BIT can detect. To enable weak peak detection for all these proteins, it is important and necessary to extend the ChIP-BIT algorithm and make it generally applicable to most ChIP-seq data.

Here we present ChIP-BIT2, an extended software package featuring the ChIP-BIT algorithm and being able to detect weak peaks across the whole genome for diverse DNA-associated proteins. ChIP-BIT2 is a C/C++ implementation and runs 40% faster than the original ChIP-BIT. We benchmarked ChIP-BIT2 on selected ChIP-seq data with experts labeled peak/nonpeak regions [14] and demonstrated that ChIP-BIT2 had a lower error rate than existing peak callers like MACS2. We have also applied ChIP-BIT2 to multiple ChIP-seq datasets downloaded from the ENCODE data portal [15] and detected binding sites of 50 proteins in the breast cancer MCF-7 cells. Results revealed that these DNA-binding proteins indeed had a different tendency to bind at promoters, enhancers, or other genomic locations, demonstrating the necessity to properly model ChIP-seq signals within a specific category of regions to better capture peaks, especially weak ones. We finally compared ChIP-BIT2 results with peaks previously identified by the ENCODE pipeline, for the same set of proteins including both TFs and HMs. At active regulatory regions in MCF-7 cells, ChIP-BIT2 recalled 92% of ENCODE peaks and in the meanwhile, it reported additional 11,813 peaks, providing more candidates for studying gene regulation in breast cancer cells [16].

## Methods

### ChIP-BIT algorithm

**Fig. 1:**
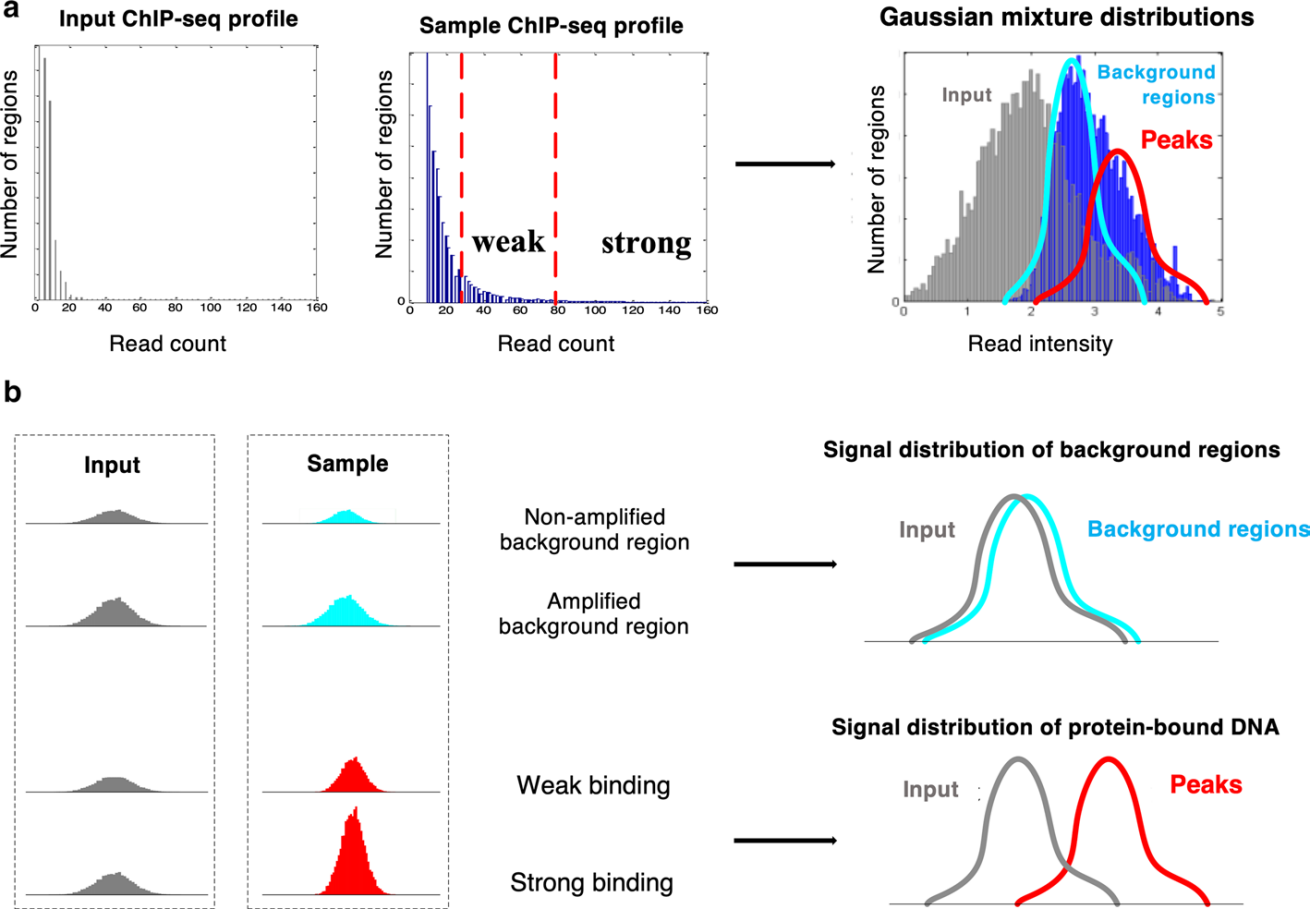
ChIP-seq peak detection using a Gaussian mixture model. ChIP-BIT2 **a** converted read counts to read intensity and then **b** used a mixture of Gaussian distributions to differentiate (strong and weak) binding events from background signals

The challenge in weak peak detection of ChIP-seq data lies in the ambiguity in differentiating weak signals of protein binding sites from noise signals produced by the background regions. In ChIP-seq data, signals from the amplified background DNAs can be as strong as true binding signals. ChIP-BIT2 shrinks the distance in read intensity distributions between strong and weak peaks using one global distribution and amplifies the difference between weak peaks and background regions using multiple local distributions. In this way, it brings more power for detecting protein binding sites with different strengths of ChIP-seq read enrichment (Fig. 1a).

To enable flexible detection for narrow or wide peaks, a sliding window is used for peak screening. The window size is adjustable to meet different resolution needs. For example, most TFs have narrow and sharp ChIP-seq peaks. A narrow window size like 50 base pairs (bps) can help identify high-resolution peak boundaries. For HMs, their peaks can be as wide as several thousand bps. A wide window size like 500 bps can effectively smooth signal fluctuation in the wide genomic region of a whole peak.

Given a ChIP-seq profile for a specific protein, assuming there were $N$ candidate genomic regions overlapping with at least two ChIP-seq reads at each, we partitioned the $n$th region into fixed-length windows and calculated read intensity $s_{n,w}$ for the window $w$. In the meanwhile, we calculated another read intensity $r_{n,w}$ using data from the matched input ChIP-seq profile. The relative distance of the window $w$ to the nearest gene TSS or enhancer center was denoted by $d_{n,w}$. ChIP-BIT2 estimated a probability for protein binding occurrence in the window $w$ of the region $n$ as [8]:

1 $$P\left( {b_{n,w} {|}s_{n,w} , d_{n,w} } \right) \propto P\left( {s_{n,w} {|}b_{n,w} } \right)P\left( {d_{n,w} {|}b_{n,w} } \right)P\left( {b_{n,w} } \right).$$

Depending on the binding or non-binding status in the variable $b_{n,w}$ (with a uniform prior on binding ‘$b_{n,w} =$ 1’ or non-binding ‘$b_{n,w} =$ 0’), we modeled $s_{n,w}$ a two-component Gaussian mixture distribution as:

2 $$\left\{ {\begin{array}{*{20}c} {P\left( {s_{n,w} {|}b_{n,w} = 1} \right) = N\left( {\mu_{1} ,\sigma_{1}^{2} } \right), } \\ {P\left( {s_{n,w} {|}b_{n,w} = 0} \right) = N\left( {r_{n,w} ,\sigma_{0}^{2} } \right).} \\ \end{array} } \right.$$

If $b_{n,w} = 1$, we assumed the region bound by the protein and modeled read intensities at protein-bound regions using a global Gaussian distribution with mean $\mu_{1}$ and variance $\sigma_{1}^{2}$, where model parameters $\mu_{1}$ and $\sigma_{1}^{2}$ were unknown and needed to be estimated. If $b_{n,w} = 0$, we assumed it a background region and modeled the read intensity using a local Gaussian distribution with mean $r_{n,w}$ and variance $\sigma_{0}^{2}$ (the variance of background signals was estimated using the input ChIP-seq data).

The second likelihood function $P\left( {d_{n,w} {|}b_{n,w} } \right)$ in Eq. (1) modeled the regulatory effects of the selected region on nearby genes. ChIP-seq data visualization around gene promoter regions (Additional file 1: Fig. S1A) and evidence from previous studies [8, 17] both suggest that: for protein binding sites, the ChIP-seq read intensity follows an exponential distribution towards the gene TSS; for background regions, the distribution is relatively uniform around the TSS. Therefore, we modeled $d_{n,w}$ a mixture distribution with two components as follows:

3 $$\left\{ {\begin{array}{*{20}c} {P\left( {d_{n,w} {|}b_{n,w} = 1} \right) = Exp\left( \lambda \right), } \\ {P\left( {d_{n,w} {|}b_{n,w} = 0} \right) = U\left( { - \frac{{d_{P} }}{2},\frac{{d_{P} }}{2}} \right).} \\ \end{array} } \right.$$

where $\lambda$ represented the exponential distribution parameter, which was unknown and needed to be estimated. $d_{P}$ represented the length of a promoter region.

For enhancers, ChIP-seq data visualization (Additional file 1: Fig. S1B) shows that the distribution of ChIP-seq read intensity is uniform and does not correlate with the distance to the enhancer center or the nearest TSS. Therefore, specifically for peak calling at distal enhancers, we assumed uniform distributions on $d_{n,w}$ as:

4 $$\left\{ {\begin{array}{*{20}c} {P\left( {d_{n,w} {|}b_{n,w} = 1} \right) = U\left( { - \frac{{d_{E} }}{2},\frac{{d_{E} }}{2}} \right),} \\ {P\left( {d_{n,w} {|}b_{n,w} = 0} \right) = U\left( { - \frac{{d_{E} }}{2},\frac{{d_{E} }}{2}} \right).} \\ \end{array} } \right.$$

where $d_{E}$ represented the length of an enhancer region.

ChIP-BIT2 used the Expectation–Maximization algorithm to iteratively estimate distribution parameters and the probability of binding occurrence in each window (Fig. 1b). Briefly, in the E-step, ChIP-BIT2 estimated the model parameters based on the inferred binding status variables ($b_{n,w}$) of all regions; in the M-step, ChIP-BIT2 updated the posterior probability $P\left( {b_{n,w} {|}s_{n,w} , d_{n,w} } \right)$ for each window using the estimated model parameters, and then updated the binding status in the variable $b_{n,w}$ accordingly. We iterated the E and M steps until the changes of parameter values were less than 5%. ChIP-BIT2 combined consecutive windows with probabilities higher than a cut-off threshold and output them together as one single peak. Depending on the protein feature and the window resolution, a sharp peak can take one or two windows and a wide peak can take more than ten windows.

### ChIP-BIT2 pipeline

**Fig. 2:**
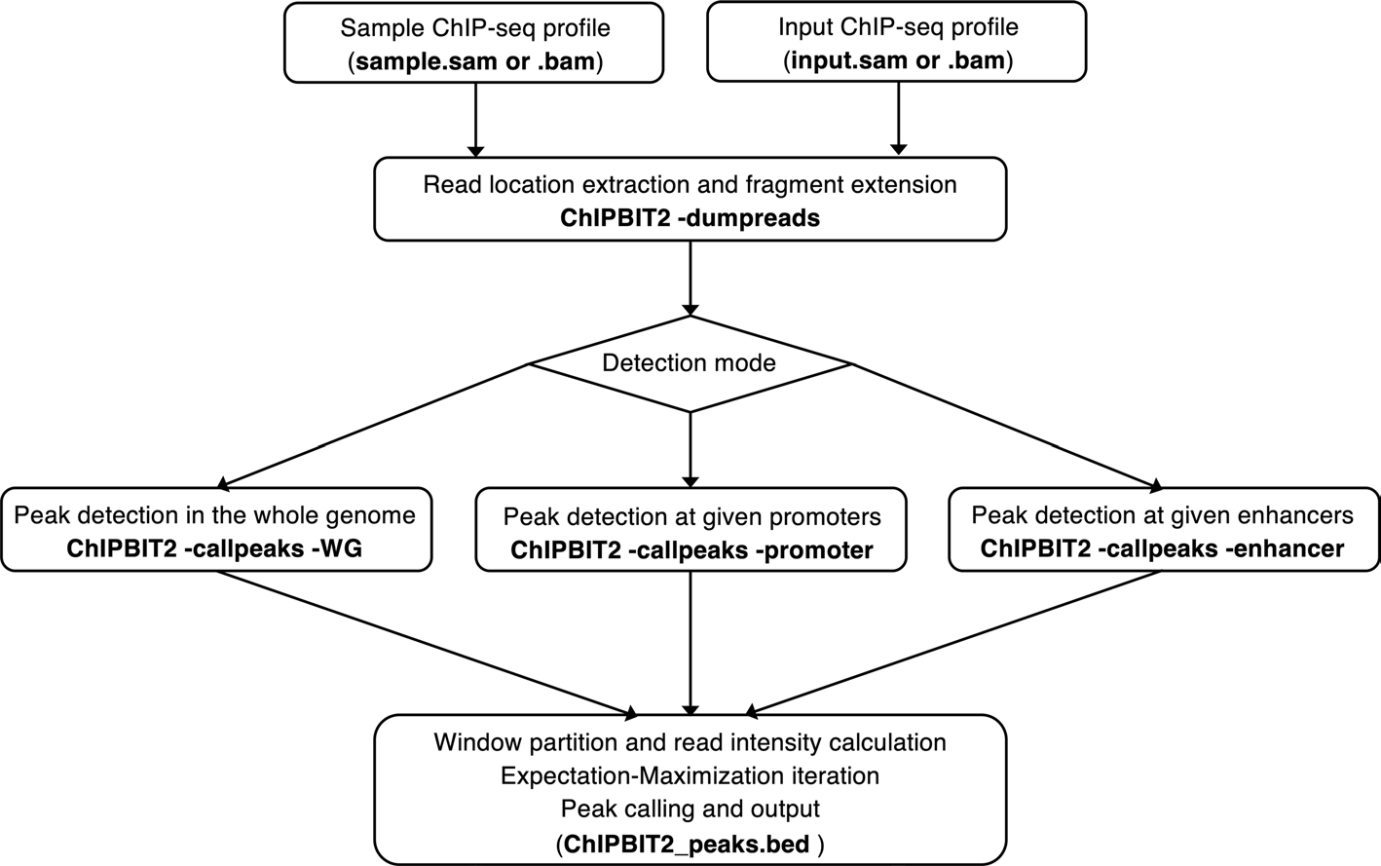
ChIP-BIT2 pipeline. ChIP-BIT2 respectively extracted read location information from sample and input ChIIP-seq SAM format profiles. Depending on the running mode, it can detect peaks from the whole genome or from annotated regulatory regions like promoters or enhancers. To enable  peak detection of different sizes, ChIP-BIT2 partitioned genomic segments into smaller windows and calculated read intensity in each window for distribution parameter learning and binding occurrence probability estimation. Windows with the posterior probability over 0.9 were output in BED format as final peaks

ChIP-BIT2 was implemented using C/C++. The pipeline of ChIP-BIT2 was shown in Fig. 2 (Additional file 1: Fig. S2). Given a pair of sample and input ChIP-seq profiles in SAM format, ChIP-BIT2 firstly extracted the genomic coordinates of individual reads from sample and input ChIP-seq profiles, respectively (Additional file 1: Fig. S3). And then it detected peaks at promoters, enhancers (if annotation files were provided), or across the whole genome.

#### Promoter mode

Promoters refer to the proximal regulatory regions centered around gene TSS. A TSS annotation file is required to enable the ‘-promoter’ running mode of ChIP-BIT2. Users can set the preferred promoter size using the ‘-s’ option. Under this mode, ChIP-BIT2 jointly modeled read intensities in the sample and input ChIP-seq profiles using the Gaussian mixture model [Eq. (2)]. In the meanwhile, it modeled the relative distance of each window to the nearest TSS using the Exponential-Uniform mixture model [Eq. (3)]. A demo of using ChIP-BIT2 for detecting peaks at promoters was provided in Additional file 1: Fig. S4.

#### Enhancer mode

Enhancers referred to distal regulatory regions interacting with promoters/TSSs in the 3D genome [18]. The linear distance of an enhancer to its target promoter/gene can be up to 1 Mbps. Some proteins like EP300, H3K27ac, and H3K4me1 specifically bind to enhancers frequently and have higher ChIP-seq signal enrichment at enhancers than at promoters or other genomic locations [10–12]. To effectively detect ChIP-seq peaks for such proteins, an enhancer annotation file is required to enable the ‘-enhancer’ running mode of ChIP-BIT2. Under this mode, ChIP-BIT2 modeled read intensities in the sample and input ChIP-seq profiles jointly using Eq. (2). Different from the promoter mode, ChIP-BIT2 modeled the relative distance of each window to the enhancer center using a Uniform distribution [Eq. (4)]. A demo of using ChIP-BIT2 for peak detection at enhancers was provided in Additional file 1: Fig. S5.

#### Whole genome mode

Promoters and enhancers are two categories of well-understood regulatory regions. There exist many other types of genomic regions also bound by DNA proteins. For example, cohesion proteins CTCF and RAD21 usually bind at the boundaries of topological associated domains and play a key role in the 3D chromatin structure [19]. Transcription initiation protein POLA2 binds to all active regulatory regions in the whole genome. For such proteins, it is important to call their ChIP-seq peaks from the whole genome, using the ‘-WG’ mode of ChIP-BIT2. As no annotated regulatory regions were needed, ChIP-BIT2 modeled read intensities from sample and input ChIP-seq profiles and predicted peaks at genome-wide locations.

## Results

### Histone modification benchmark analysis

The ChIP-BIT algorithm has been benchmarked on narrow TFBSs and demonstrated to perform better than conventional peak callers [8, 20]. To evaluate the accuracy of ChIP-BIT2 on detecting narrow or wide histone modifications, for selected HMs we benchmarked ChIP-BIT2 on experts-labeled peak/nonpeak regions. We used an HM benchmark dataset [14] including 10,253 H3K4me3 regions (narrow pattern) and 2573 H3K36me3 regions (broad pattern), the protein binding statuses of which were respectively and independently labeled by three experts by visualizing ChIP-seq data across multiple immune cell samples (T-cell, B-cell, and monocyte). For some peaks with low-resolution peak boundaries, peak-start and -end regions were respectively labeled.

Here we compared the detection accuracy of ChIP-BIT2 to that of MACS2 (2020.4 version) [21] and CNN-Peaks [22]. MACS2 was widely used in ChIP-seq peak detection. It featured regions with high read counts as peaks so most of its detected peaks were strong. CNN-Peaks was a supervised machine-learning approach, not making distribution assumptions on read depth but learning proper cut-off thresholds at labeled regions from the sample ChIP-seq data. As CNN-Peaks used different thresholds to determine the peak/nonpeak status for regions with different ChIP-seq read depths, it could capture weak binding events.

We downloaded H3K4me3 and H3K36me3 ChIP-seq data and their matched input in K562 and GM12878 cells, from ENCODE data portal (https://www.encodeproject.org/) [15]. K562 and GM12878 cell lines are both blood-specific, providing a matching context to the benchmark data. In total, four ChIP-seq datasets and three peak calling tools were included in this comparison. Peak detection errors were assessed using PeakError [14]. To account for both false positives and false negatives, we calculated the F1 score, the harmonic mean of precision and recall (2 * precision * recall/(precision + recall)).

To fairly compare the peak detection accuracy between supervised (CNN-Peaks) and unsupervised approaches (ChIP-BIT2 and MACS2), we performed 4-fold cross-validation: using three folds to optimize model parameters of each method and using the hold out one fold to assess detection accuracy. Under this setting, the difference between the three selected methods was small but ChIP-BIT2 had the highest F-1 score (Table 1). In reality, in a ChIP-seq profile, the peak regions were largely unknown before peak detection analysis. The model parameters of a peak caller cannot be specifically optimized using signals from true peak/nonpeak regions. This largely limited the application of supervised approaches. Using pre-trained models to predict peaks in a new ChIP-seq profile may not return good results either,  because ChIP-seq experiments were highly context-specific.

**Table 1:** F1-score and run-time of competing peak callers on H3K4me3 and H3K36me3 benchmark region detection using ENCODE ChIP-seq datasets

Cell line	K562		GM12878	
Histone protein	H3K4me3	H3K36me3	H3K4me3	H3K36me3
F1-score (Supervised)				
ChIP-BIT2	0.93	0.90	0.95	0.90
MACS2	0.89	0.78	0.93	0.83
CNN-peaks	0.91	0.85	0.90	0.88
F1- score (unsupervised)				
ChIP-BIT2	0.88	0.82	0.91	0.82
MACS2	0.82	0.77	0.84	0.79
Run-time (unsupervised)				
ChIP-BIT2	14m1s	9m21s	15m7s	9m9s
MACS2	3m42s	2m39s	5m35s	2m32s

Both ChIP-BIT2 and MACS2 were unsupervised approaches. As shown in Table 1, under unsupervised settings, ChIP-BIT2 had a higher accuracy than MACS2. ChIP-BIT2 ran reasonably fast (< 15 mins) on DELL T7600 workstation with 3.1 GHz CPU (32 cores) and 128 GB RAM. As ChIP-BIT2 detected additional weak binding events by screening many more candidate regions, its run-time was slightly longer than MACS2.

### Run-time of ChIP-BIT2

**Fig. 3:**
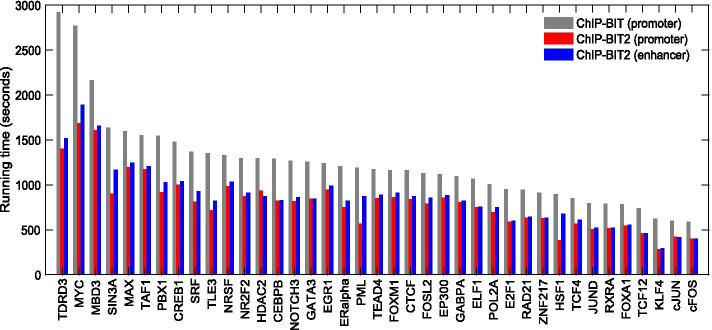
Running time comparison between ChIP-BIT2 and ChIP-BIT

To evaluate the running time of ChIP-BIT2 in different scenarios, we downloaded ChIP-seq data of 39 TFs in breast cancer MCF-7 cells and their matched input data from ENCODE and the GEO databases (Additional file 1: Table S1). We also downloaded TSS and enhancer annotation files for MCF-7 cells from the SCREEN webserver (https://screen.encodeproject.org/) [23]. In total, we obtained 25,802 promoters (possibly overlapping for closely located genes) and 34,599 enhancers. ChIP-BIT2 and ChIP-BIT were respectively applied to individual ChIP-seq datasets, under CentOS Linux 7.3 system, on a DELL T7600 workstation with 3.1 GHz CPU (32 cores) and 128 GB RAM. ChIP-BIT2 achieved a speed improvement of ~ 40% over ChIP-BIT (Fig. 3). Moreover, although the number of enhancers was 30% more than the number of promoters, ChIP-BIT2 had a similar running time between ‘promoter’ and ‘enhancer’ modes.

### DNA proteins exhibiting different binding tendency to promoters or enhancers

**Fig. 4:**
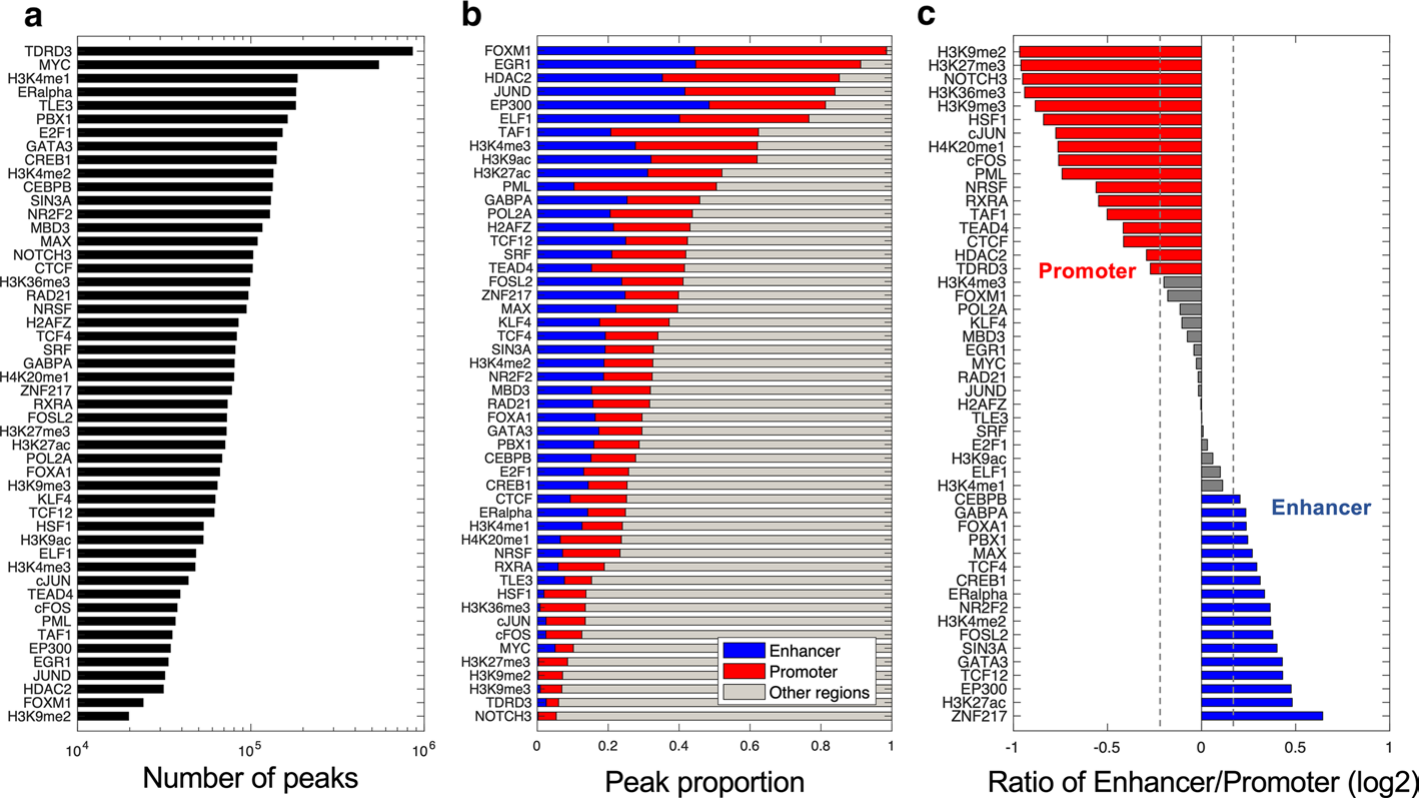
Peak detection summary of ChIP-BIT2 for 50 DNA proteins. **a** Using breast cancer MCF-7 cells ChIP-seq data of 39 TFs and 11 HMs from ENCODE data portal, ChIP-BIT2 detected peaks from the whole genome. **b** We calculated the proportion of peaks detected from promoters, enhancers or at other regions (peaks from whole genome minus peaks in promoters or enhancers), respectively, and **c** calculated the log2 ratio of the numbers of peaks between enhancers and promoters

We applied ChIP-BIT2 to another 11 HMs with available MCF-7 ChIP-seq data in the ENCODE data portal (Additional file 1: Table S1). The number of peaks for each of the above TFs and these HMs was shown in Fig. 4a. For TFs like MYC and ER-alpha that were reported to be highly active in MCF-7 cells [24–26], ChIP-BIT2 detected a high number of peaks. Further, for each protein, we calculated the proportion of its ChIP-seq peaks in annotated promoters, enhancers, or the other regions (Fig. 4b) and also calculated the ratio between promoter-overlapping peaks and enhancer-overlapping peaks (Fig. 4c).

For enhancer marker proteins like EP300 and H3K27ac, their ChIP-BIT2 detected peaks were significantly more enriched at enhancers than at promoters (fold change 1.4; *p* value < 0.01, fisher exact test). NOTCH3 has been previously demonstrated to bind to promoters of breast cancer genes [8]. Indeed, we detected twofold more NOTCH3 binding events at promoters than at enhancers (fold change 2; *p* value < 0.001, fisher exact test). Transcription initiation protein POL2A usually bind to transcriptional regulatory regions. As expected, we observed similar proportions of POL2A ChIP-seq peaks between promoters and enhancers. There were also proteins with binding sites mostly located outside the annotated promoters or enhancers (Fig. 4b, grey color). For example, CTCF functions as an insulator and bind at the topological associating domains boundary area [27]. In our analysis, only a small proportion (25%) of CTCF peaks overlapped with existing promoters or enhancers. Therefore, to efficiently call ChIP-seq peaks, we recommended running ChIP-BIT2 in a proper mode if prior knowledge of the binding preference of the protein was available.

### Large-scale application to breast cancer MCF-7 cell line data

**Fig. 5:**
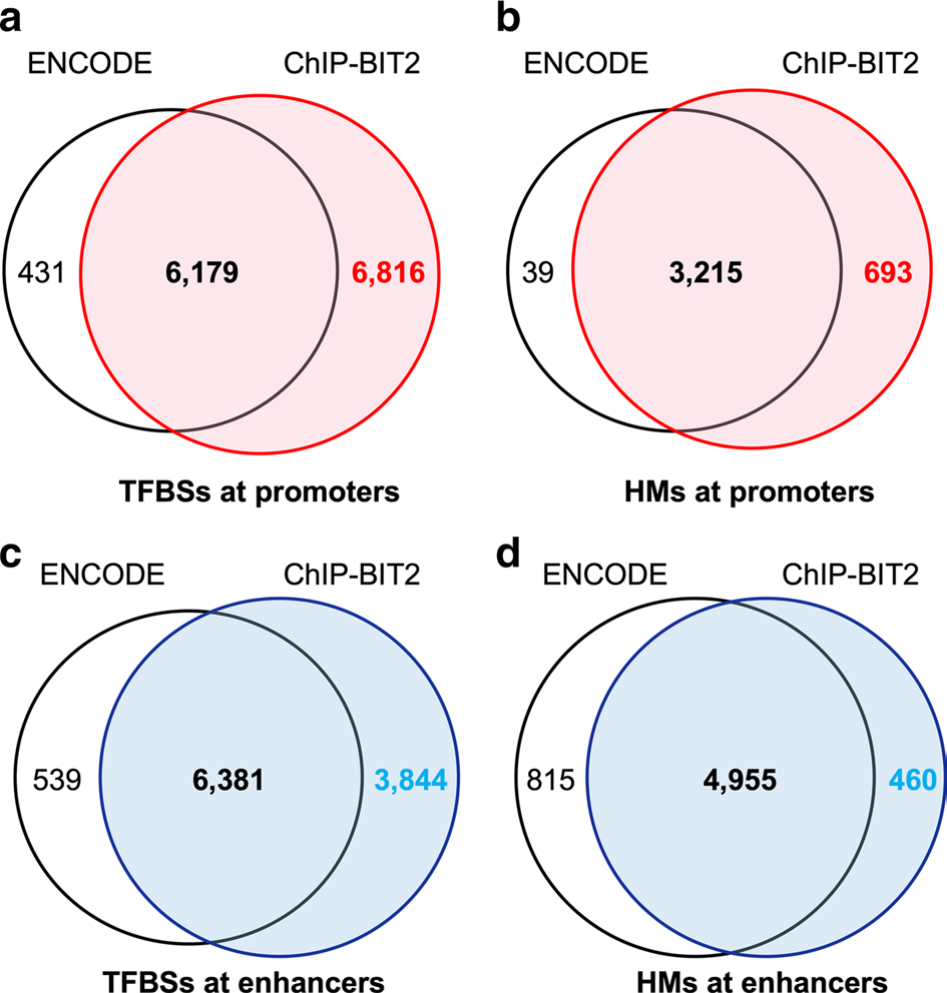
Venn diagram of binding events detected by ChIP-BIT2 and ENCODE at MCF-7 active promoters or enhancers. **a** TFBSs at 489 promoters; **b** HMs at 489 promoters; **c** TFBSs at 1050 enhancers; **d** HMs at 1050 enhancers

To demonstrate that ChIP-BIT2 detected additional peaks that were functionally important but missed by conventional peak callers, for the same set of proteins, we compared ChIP-BIT2 results to peaks detected by the ENCODE pipeline (Additional file 1: Table S1; 26 TFs and 11 HMs). We focused our comparison to regulatory regions associated with ‘actively’ expressed genes in MCF-7 cells because peaks in these regions were more likely to be functional (having regulatory effects). To identify active promoters or enhancers in MCF-7 cells, we downloaded two RNA-seq datasets from the GEO database (accession numbers: GSE62789 and GSE51403). 489 genes were significantly (adjusted *p* value < 0.05) and actively (log2FC > 1) expressed in both datasets. Regions (± 10 kbps) around TSSs of these 489 genes were selected as ‘active’ promoters. 1050 enhancers looping with the above promoters through ENCODE MCF-7 cell line ChIA-PET 3D chromatin interactions were selected as ‘active’ enhancers. Venn diagram of ChIP-BIT2 peaks and ENCODE peaks overlapping with these selected active regulatory regions were shown in Fig. 5. Overall, ChIP-BIT2 recalled 92% of ENCODE peaks and identified additional 11,813 (52%) new peaks.

Specifically, for in total of 26 TFs, ChIP-BIT2 recovered ~ 93% (6179/6610) ENCODE peaks in promoters and predicted 6816 new peaks. For specific proteins, for example, few peaks were identified for TDRD3 in the ENCODE database. After evaluating TDRD3 read intensities using ChIP-BIT2, we found that its read enrichment at many regions in the sample ChIP-seq profile was much higher than that in the input data. Therefore, ChIP-BIT2 finally detected TDRD3 peaks in 438 promoter regions, covering nearly 90% of the selected active promoters. For MBD3, ENCODE peaks were on 35% of promoters while ChIP-BIT2 detected more peaks on 55% of promoters. For all 11 histone proteins, ChIP-BIT2 captures nearly all ENCODE peaks (99%, 3215/3254), demonstrating its strong capability of detecting wide histone modifications. An additional set of 693 histone modifications were captured, ~ 20% in ChIP-BIT2 results. A similar comparison was done for peaks at MCF-7 active enhancers. For TFs, ChIP-BIT2 identifies 10,225 peaks, overlapping with 92% ENCODE peaks and providing 3844 new peaks. For HMs, the similarity between the two peak calling approaches was also high, with an overlap rate of 86%. In summary, for both TFs and HMs, ChIP-BIT2 detected a majority of peaks identified by the ENCODE pipeline and also predicted new peaks at functionally important regulatory regions.

## Discussion

ChIP-BIT2 can detect strong and weak peaks from annotated regulatory regions or the whole genome, using a Bayesian model to integrate sample and input ChIP-seq profiles. To better capture ChIP-seq peaks at regulatory regions, ChIP-BIT2 takes into account protein binding locations when it estimates the probability of each peak because a weak peak locating closer to the gene TSS could have a higher regulatory effect on that gene than peaks located farther. We demonstrated the accuracy and wide applicability of ChIP-BIT2 using benchmark data and public data in the ENCODE and GEO databases.

Currently, ChIP-BIT2 detects peaks from the given ChIP-seq data one at a time. We are working on its parallel mode to enable peak calling from multiple ChIP-seq profiles together, facilitating the robust peak calling using multiple replicates of one protein or association analysis between multiple proteins. Currently, ATAC-seq data are widely used to capture open chromatin regions in a particular tissue or cell type [28, 29]. Different from ChIP-seq data, ATAC-seq used paired-end reads. Yet, some existing peak callers simply treat the paired ends of one long read as two separate single-end reads and then detect peaks in the same way as from a ChIP-seq profile. This simplification may break some ultra-wide open chromatin regions into several disconnected narrow peaks, causing errors in the genome-wide chromatin accessibility analysis. We plan to extend the preprocessing function of ChIP-BIT2 and enable modeling read intensity in ATAC fragments of different lengths. With such an extension, ChIP-BIT2 will be able to detect ATAC-seq peaks.

## Conclusions

We have developed a C++ software package, ChIP-BIT2, for DNA–protein binding site detection from the ChIP-seq data. ChIP-BIT2 can capture the subtlety in differentiating weak binding sites from background regions by jointly modeling read intensities from sample and input ChIP-seq profiles. ChIP-BIT2 exhibits an accurate performance on detecting both narrow and wide ChIP-seq peaks and it has a broad applicability in TF or HM ChIP-seq data analysis.

## Supplementary Information

**Additional file 1**. ChIP-BIT2 demo instructions, Figures S1–S5 and Table S1.

## Data Availability

The datasets supporting the conclusions of this article are available in the ENCODE data portal (https://www.encodeproject.org/, Table s1) and ncbi geo database (https://www.ncbi.nlm.nih.gov/gds): gSE26831, GSE41561, GSE38901, GSE44737, GSE28008, GSE22612, and GSE62789. ChIP-BIT2 package is implemented using C/C++ under Linux environment and is publicly accessible at http://sourceforge.net/projects/chipbitc/.

## Abbreviations

ATAC-seq: Assay for Transposase-Accessible Chromatin using sequencing
ChIP-BIT2: Bayesian inference of target genes using ChIP-seq data V2
ChIP-seq: Chromatin Immunoprecipitation Sequencing
ChIA-PET: Chromatin Interaction Analysis by Paired-End Tag Sequencing
ENCODE: Encyclopedia of DNA elements
F1: Harmonic mean of precision and recall
HM: Histone modification
MACS2: Model-based analysis of ChIP-seq V2
TF: Transcription factor
TFBS: Transcription factor binding site
TSS: Transcription starting site
