# Supplementary material for: ChIP-BIT2: a software tool to detect weak binding events using a Bayesian integration approach
Source: BMC Bioinformatics. 2021 Apr 15;22:193. doi: 10.1186/s12859-021-04108-5 (PMC8051094; doi:10.1186/s12859-021-04108-5)
Supplement: Supplementary file 1 — Additional file 1. ChIP-BIT2 demo instructions, Figures S1–S5 and Table S1. [file 12859_2021_4108_MOESM1_ESM.pdf]

Additional file 1

ChIP-BIT2 usage

Preprocessing: The usage of ChIP-BIT2 is shown in **Fig. S2**. ChIP-BIT2 first extracts read location information from SAM format ChIP-Seq profile (**Fig. S3**). It processes the reads chromosome by chromosome. If only BAM file is provided, samtools (http://www.htslib.org/) is needed to convert BAM file to SAM file.

Peak calling: ChIP-BIT2 can be run in 'promoter mode' (-promoter option; see **Fig. S4** for an example) or 'enhancer mode' (-enhancer option; see **Fig. S5** for an example) with annotation file provided (-a option), or the whole genome mode similar to the use of other peak callers. Promoter region is a certain genomic area surrounding each transcription starting site (TSS), which can be obtained from reference genome files (e.g., UCSC RefSeq file from https://genome.ucsc.edu/index.html). Enhancer regions can be obtained from the ENCODE SCREEN server (https://screen.encodeproject.org/) or FANTOM5 database (https://fantom.gsc.riken.jp/5/datafiles/latest/extra/Enhancers/). ChIP-BIT2 identified peaks within a certain area around each annotated region (-s option). Two additional parameters including the probability cut-off threshold for output (-p 0.9) and the number of EM iterations (-EM 100) can be adjusted to meet different needs.

Results: ChIP-BIT2 peak outputs are similar under different modes. Besides reporting genome coordinates of detected peaks, it also outputs read intensities and the probability for each peak.

promoter mode

Peak_ID, Chr, Point1, Point2, Sample_read_intenisty, Input_read_intenisty, Probability, Gene_symbol

enhancer mode

Peak_ID, Chr, Point1, Point2, Sample_read_intenisty, Input_read_intenisty, Probability, Enhancer_ID

whole genome mode

Peak_ID, Chr, Point1, Point2, Sample_read_intenisty, Input_read_intenisty, Probability

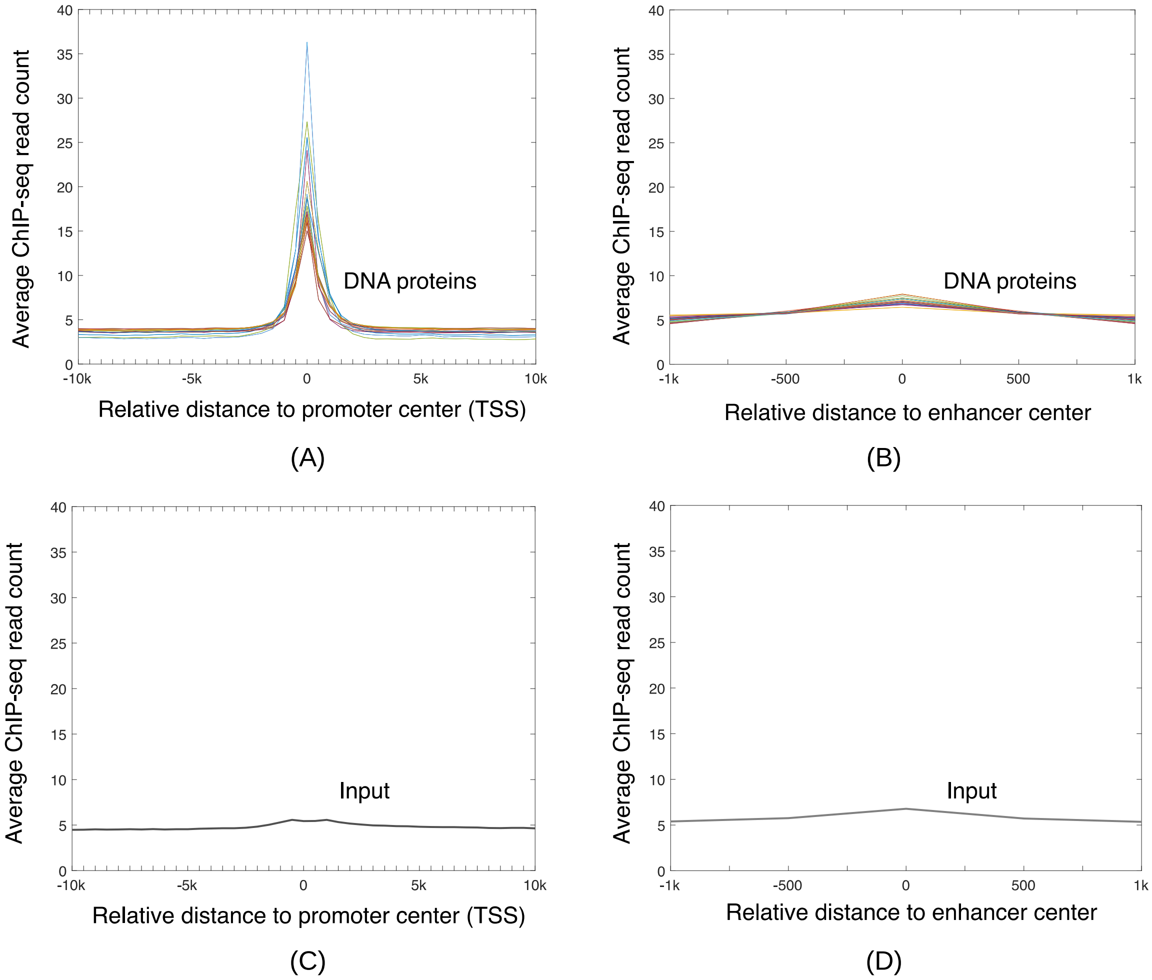

Fig. S1. ChIP-seq read enrichment at genomic locations around promoter (TSS) and enhancers. (A) and (B) Each color line represents a ChIP-seq profile of one DNA-protein in breast cancer MCF-7 cells. (C) and (D) One input ChIP-seq profile for breast cancer MCF-7 cells.

```
        USAGE:
        Options:
        ./ChIPBIT -dumpreads [Input format ('SAM')] [Path to the file that contains mapped tag data ('stdin' for piping)] [output
 directory for parsed reads]
        or
        ChIPBIT -callpeaks -promoter | -enhancer (this must be specified right after -callpeaks option)
        -n Experiment_name
        -t Sample ChIP seq file after preprocessing
        -c Input ChIP seq file after preprocessing
        -m mappability file
        -a promoter or enhancer annotation file
        -s (optional)searching scale around TSS or center of enhancer, default +-10k around TSS or +-1k around enhancer center
        -w (optional)partition window size, default 200 bps
        -p (optional)probability threshold, default 0.9
        -EM (optional)Number of EM iterations, default 100
cbil@cbil-Precision-WorkStation-T5500:/media/Disk1_/ChIP_BIT_resources/MCF7_JHU/MCF7_ChIPSeq/ChIPBIT2$
```

Fig. S2. The usage message for executing the ChIP-BIT2 program.

```
ChIPBIT -dumpreads SAM MCF7_NOTCH3.sam Sample_read
Preprocessing:
input format: SAM
chip_seq_eland_op_fp: MCF7_NOTCH3.sam
parsed_reads_op_dir: Sample_read
Added 3
Added 6
Added 8
Added 15
Added 4
Added 16
Added 2
Added 1
Added 7
Added 14
Added 9
Added X
Added 11
Added 12
Added 18
Added 10
Added 17
Added 13
Added 5
Added 20
Added 19
Added 22
Added 21
Added M
Added Y
```

Fig. S3. Read location information extracted from the SAM formatted ChIP-seq data.

```
 ./ChIPBIT -callpeaks -promoter -n NOTCH3 -t Sample_reads -c Input_reads -m hg19_mappability_20k.txt -
a hg19_RefSeq.txt -s 10000 -w 200 -p 0.9 -EM 100
Experimental ID:                  NOTCH3
Sample ChIP-seq read path:        Sample_reads
Input ChIP-seq read path:         Input_reads
Mappability map file:             hg19_mappability_20k.txt
Annotation file:                  hg19_RefSeq.txt
Promoter region scale around TSS:       +-10000.0 bps
Window size:    200.000000 bps
Posterior probability threshold:        0.90
EM iteration:   100 rounds
processing Chr3...
processing Chr6...
processing Chr8...
processing Chr15...
processing Chr4...
processing Chr16...
processing Chr2...
processing Chr1...
processing Chr7...
processing Chr14...
processing Chr9...
processing ChrX...
processing Chr11...
processing Chr12...
processing Chr18...
processing Chr10...
processing Chr17...
processing Chr13...
processing Chr5...
processing Chr20...
processing Chr19...
processing Chr22...
processing Chr21...
processing ChrM...
processing ChrY...
```

(A)

```
ChIP-BIT process starts!
......
Number of candidate ChIP-seq regions: 194336
......
Load transcript or gene annotation file!
......
Number of transcripts: 49392
......
Number of unique annotated regions: 25802
......
Genome partition: 200
......
Calculate read intensity of each window!
......
Number of candidate windows: 30711
......
......
EM iteraction to estimate parameters and posterior probability of each window!
TFBS_lambda: 0.550722
Prior probability of 'foreground' TFBS: 0.393456
Prior probability of 'background' events: 0.606544
TFBS_mean: 2.78982
TFBS_variance: 0.181945
Background_mean: 1.90203
Background_variance: 0.693577
Probability_threshold: 0.9
......
Write ChIP-BIT results to file!
......
Merge 629 significant ChIP-BIT windows to peaks!
......
581 significant peaks!
```

(B)

Fig. S4. ChIP-BIT2 peak detection at gene promoter regions. (A) Input data normalization and candidate region searching considering genome mappability; (B) ChIP-BIT2 model parameter estimation and peak detection.

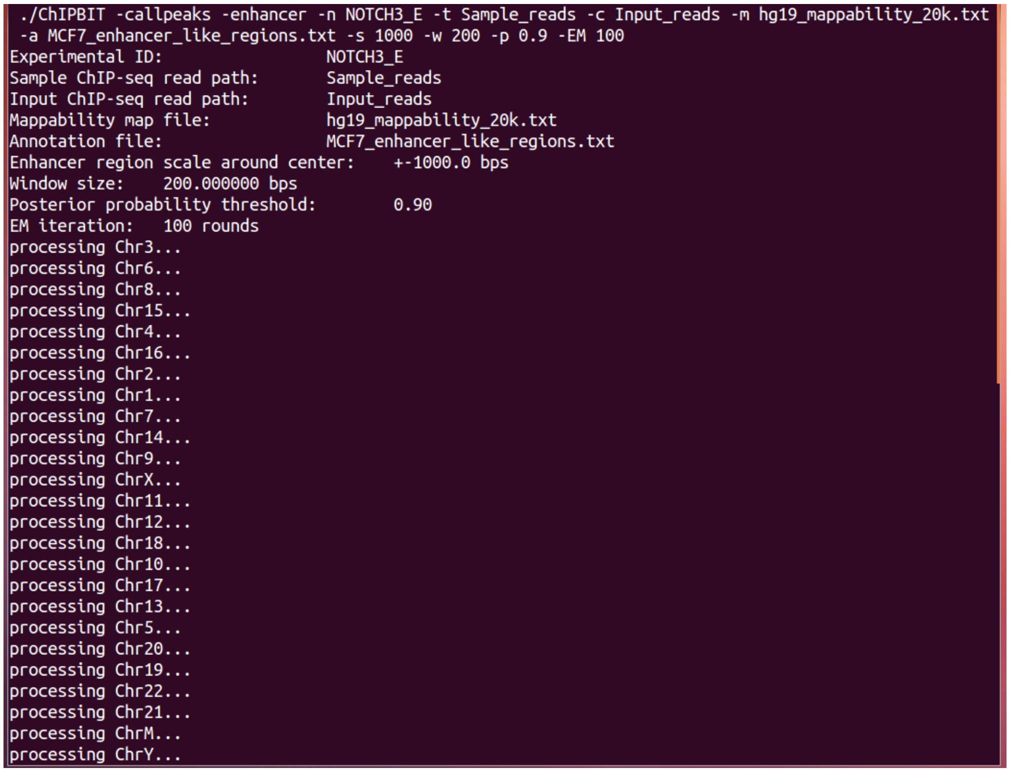

```
./ChIPBIT -callpeaks -enhancer -n NOTCH3_E -t Sample_reads -c Input_reads -m hg19_mappability_20k.txt
-a MCF7_enhancer_like_regions.txt -s 1000 -w 200 -p 0.9 -EM 100
Experimental ID:                NOTCH3_E
Sample ChIP-seq read path:      Sample_reads
Input ChIP-seq read path:       Input_reads
Mappability map file:           hg19_mappability_20k.txt
Annotation file:                MCF7_enhancer_like_regions.txt
Enhancer region scale around center:    +-1000.0 bps
Window size:    200.000000 bps
Posterior probability threshold:        0.90
EM iteration:   100 rounds
processing Chr3...
processing Chr6...
processing Chr8...
processing Chr15...
processing Chr4...
processing Chr16...
processing Chr2...
processing Chr1...
processing Chr7...
processing Chr14...
processing Chr9...
processing ChrX...
processing Chr11...
processing Chr12...
processing Chr18...
processing Chr10...
processing Chr17...
processing Chr13...
processing Chr5...
processing Chr20...
processing Chr19...
processing Chr22...
processing Chr21...
processing ChrM...
processing ChrY...
```

(A)

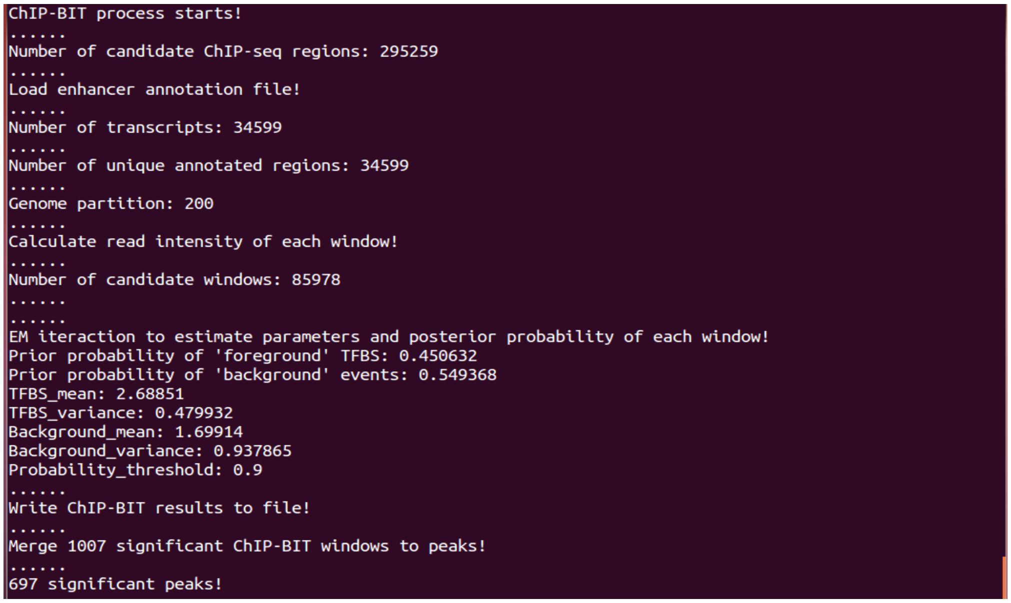

```
ChIP-BIT process starts!
......
Number of candidate ChIP-seq regions: 295259
......
Load enhancer annotation file!
......
Number of transcripts: 34599
......
Number of unique annotated regions: 34599
......
Genome partition: 200
......
Calculate read intensity of each window!
......
Number of candidate windows: 85978
......
......
EM iteraction to estimate parameters and posterior probability of each window!
Prior probability of 'foreground' TFBS: 0.450632
Prior probability of 'background' events: 0.549368
TFBS_mean: 2.68851
TFBS_variance: 0.479932
Background_mean: 1.69914
Background_variance: 0.937865
Probability_threshold: 0.9
......
Write ChIP-BIT results to file!
......
Merge 1007 significant ChIP-BIT windows to peaks!
......
697 significant peaks!
```

(B)

Fig. S5. ChIP-BIT2 peak detection at enhancer regions. (A) Input data normalization and candidate region searching considering genome mappability; (B) ChIP-BIT2 model parameter estimation and peak detection.

Table S1. MCF7 ChIP-seq profiles from ENCODE and GEO database.

Data source	Protein name
ENCODE	CEBPB, CTCF, E2F1, EGR1, ELF1, EP300, FOSL2, FOXM1, GABPA, GATA3, HDAC2, JUND, MAX, MYC, NR2F2, NRSF, PML, POLR2A, RAD21, SIN3AK20, SRF, TAF1, TCF7, TCF12, TEAD4, ZNF217
GSE26831	c-FOS, c-JUN, FOXA1
GSE41561	CREB1, ER-α, KLF4, RXRA, TLE3
GSE38901	HSF1
GSE44737	MBD3
GSE28008	PBX1
GSE22612	TDRD3
In house	NOTCH3
ENCODE	H2AFZ, H3K27ac, H3K27me3, H3K36me3, H3K4me1, H3K4me2, H3K4me3, H3K9ac, H3K9me2, H3K9me3, H4K20me1
